# Outcomes of lung transplantation for chronic obstructive pulmonary disease

**DOI:** 10.5578/tt.20239703

**Published:** 2023-09-22

**Authors:** S. TÜRKKAN, F. ÇELİK BAŞARAN, M.F. ŞAHİN, M.A. BEYOĞLU, M. BİNDAL, A. YAZICIOĞLU, E. YEKELER

**Affiliations:** 1 Clinic of Thoracic Surgery and Lung Transplantation, Ankara Bilkent City Hospital, Ankara, Türkiye; 2 Clinic of Anesthesiology and Reanimation, Ankara Bilkent City Hospital, Ankara, Türkiye

**Keywords:** chronic lung allograft disfunction, COPD, lung transplantation, primary graft disfunction

## Abstract

**ABSTRACT:**

Outcomes of lung transplantation for chronic obstructive pulmonary disease

**Introduction:**

Chronic obstructive pulmonary disease is a progressive airway disease that can progress to the terminal stage requiring oxygen supply. In this period, lung volume reduction therapies and/or lung transplantation may be considered. Morbidity and mortality risks due to transplant surgery and posttransplant immunosuppressive therapy show the importance of selecting the best candidates who will benefit from transplantation. In this context, BODE index criteria serve as important markers. This study aimed to analyze the outcomes of lung transplantation in patients with chronic obstructive pulmonary disease and to identify variables that may affect post-transplant clinical outcomes.

**Materials and Methods:**

Lung transplants diagnosed with chronic obstructive pulmonary disease performed in our center between March 2013 and January 2023 were included in the study. Demographic information and both pre-op and post-op clinical data of the transplant patients were collected. The effect of BODE index criteria and other pre-transplant clinical data on short- and long-term outcomes after transplantation were investigated.

**Results:**

During the study period, 34 lung transplants were performed for chronic obstructive pulmonary disease. One patient died during the operation, three patients received single transplants, and 30 received double transplants. Post-operative primary graft dysfunction was more common in single transplant recipients. The results were comparable between single and double transplants in terms of post-transplant pulmonary function and the development of chronic lung allograft dysfunction. BODE index criteria had no effect on early and late post-operative clinical data, however intra-operative use of extracorporeal membrane oxygenation, primary graft dysfunction, and prolonged extubation were significantly higher in recipients younger than 60 years.

**Conclusion:**

Our study suggests that prelisting demographic and clinical data of chronic obstructive pulmonary disease patients had no significant effect on post-operative outcomes, however, intra-operative ECMO use, prolonged extubation, primary graft dysfunction (p< 0.05 for each) and chronic rejection (p> 0.05) were more common in patients who are <60 years of age. These data need to be confirmed by larger studies.

## Abstract (tr)

**ÖZ:**

Kronik obstrüktif akciğer hastalığında akciğer nakli sonuçları

**Giriş:**

Kronik obstrüktif akciğer hastalığı, tüm dünyada morbidite ve mortalitesi artan obstrüktif bir hava yolu hastalığıdır. Hastalık uzun süreli oksijen tedavisi gerektiren terminal döneme kadar ilerleyebilir. Bu dönemde hastalarda akciğer hacmi azaltıcı tedaviler ve/veya akciğer nakli düşünülebilir. Nakil cerrahisinin morbidite ve mortalite risklerinin yanında transplant sonrası uygulanacak immünosupresif ilaçların muhtemel birçok klinik yan etkisi, akciğer naklinden fayda sağlayacak en iyi nakil adayının seçilmesinin önemini göstermektedir. Bu bağlamda BODE indeksi kriterleri önemli belirteçtir. Bu çalışmanın amacı, kronik obstrüktif akciğer hastalığı hastalarında akciğer nakli sonuçlarını analiz etmek ve transplant sonrası klinik sonuçları etkileyebilecek değişkenleri belirlemekti.

**Materyal ve Metod:**

Merkezimizde Mart 2013-Ocak 2023 tarihleri arasında gerçekleştirilen kronik obstrüktif akciğer hastalığı tanılı akciğer nakilleri çalışmaya dahil edildi. Transplant hastalarının demografik bilgileri ve hem pre-op hem post-op klinik verileri toplandı. BODE indeksi kriterleri başta olmak üzere diğer pre-transplant klinik verilerin transplant sonrası kısa ve uzun dönem sonuçlara etkisi araştırıldı.

**Bulgular:**

Çalışma süresi boyunca kronik obstrüktif akciğer hastalığı nedeniyle 34 akciğer nakli yapıldı. Bir olgu intraoperatif kaybedildi, üç tanesi tek, 30 tanesi çift transplant alıcısıydı. Post-operatif primer greft disfonksiyonu single transplant alıcılarında anlamlı olarak fazla idi. Tek ve çift transplantlar arasında post transplant pulmoner fonksiyonlar ve kronik rejeksiyon gelişmesi açısından benzer sonuçlar vardı. BODE indeksi kriterlerinin post operatif erken ve geç dönem klinik verilere etkisi gözlemlenmezken, 60 yaş altı alıcı- larda intraoperatif ekstrakorporeal membran oksijenasyonu kullanımı, primer greft disfonksiyonu ve uzamış ekstübasyon durumu anlamlı olarak fazla idi.

**Sonuç:**

Çalışmamız listeleme sırasında kronik obstrüktif akciğer hastalığı olgularının sahip olduğu belirli demografik ve klinik verilerin post-op sonuçlara anlamlı etkisinin olmadığını, ancak 60 yaş altı grupta ilginç olarak intraoperatif ekstrakorporeal membran oksijenasyonu kullanımı, gecikmiş ekstübasyon, primer greft disfonksiyonu (her biri için p< 0,05) ve kronik rejeksiyonun (p> 0,05) daha fazla görüldüğünü ileri sürmektedir. Verilerin daha geniş katılımlı çalışmalarla desteklenmesi gerekmektedir.

## INTRODUCTION

Chronic obstructive pulmonary disease (COPD) is an obstructive airway disease with increasing morbidity and mortality worldwide. While the primary cause is typically attributed to cigarette smoke and occupational exposure, a small percentage of cases may be attributed to genetic predisposition, specifically alpha-1 antitrypsin deficiency. In addition to first-line interventions for the disease, including smoking cessation, medical treatment, and pulmonary rehabilitation, the condition can eventually progress to a terminal stage marked by a reliance on oxygen supplementation. During this phase, patients may be evaluated for bronchoscopic or surgical lung volume reduction treatments, as well as lung transplantation ( 1 , 2 ).

COPD held the status of the most prevalent indication for lung transplantation until 2005 when the lung allocation score (LAS) was incorporated to establish the transplant priority for candidates in North America and the Eurotransplant region. Idiopathic pulmonary fibrosis has been the most common indication since the use of LAS, in which candidates with high scores were prioritized ( 3 , 4 , 5 ).

Some authors have noted a surveillance period of around six years for patients with Global Strategy for the Diagnosis, Management, and Prevention of Chronic Obstructive Pulmonary Disease (GOLD) Class 3 and 4 (COPD). Severe morbidity and mortality risks that may be caused by transplant surgery and the vulnerability to many complications caused by immunosuppressive drugs show the importance to select the best transplant candidate to benefit from a lung transplant ( 6 , 7 ).

Forced expiratory volume in one second (FEV_1_) has been used as a marker to determine the severity of COPD. However, Celli et al. stated that FEV_1_ alone is not sufficient as an indicator to determine the severity of the disease and survival in COPD cases. For this purpose, they established a scoring system called BODE. BODE represents a combination of four variables; body-mass index (B); airflow obstruction (O); dyspnea (D); and exercise capacity (E) ( 8 , 9 , 10 ) ( Table 1 ). In accordance with this scoring system, as patients receive higher scores, the severity of the disease escalates, leading to a decrease in life expectancy. Today, transplant candidates with COPD, who are expected to experience increased life expectancy and enhanced quality of life, are selected based on this scoring system ( 11 ).

The objective of this study is to assess the outcomes of lung transplantation in patients with COPD by comparing the results of single and double transplants in terms of both short-term and long-term effects. Secondly, we aimed to explore the impact of BODE index components on the clinical trajectory after transplantation.

**Table 1:** The BODE index scale

Parameter/scoring	0	1	2	3
Body mass index (kg/m^2^)	>21	≤21		
Obstruction (FEV_1_)	≥65%	50-64%	35-49%	<35%
Dyspnea (mMRC)	0-1	2	3	4
Exercise capacity (6-minute walking test)	≥350 m	250-349 m	150-249 m	≤149 m

FEV_1_: Forced expiratory volume in one second, mMRC: The modified medical research council dyspnea scale.

## MATERIALS and METHODS

This is a retrospective observational study conducted at a single center. The study included lung transplant cases with a diagnosis of COPD that were performed at the Türkiye Yüksek İhtisas Hospital and Ankara Bilkent City Hospital Lung Transplant Clinic from March 2013 to January 2023. The required approval for the study was obtained from Ankara Bilkent City Hospital Local Ethics Committee with ID number E1-2022-3134. The recipients’ age, gender, and transplant indication were recorded. Demographic data such as pulmonary function tests, comorbidities, height, weight, body mass index (BMI), 6-minute walking test (6MWT) distances and modified medical research council dyspnea scale were obtained. Pulmonary artery pressure at the listing time and the LAS and BODE score calculated at the last pretransplant outpatient clinic visit were also recorded.

The operation dates and last visit dates of the patients who underwent lung transplantation were recorded. Cases followed for less than one month were excluded from the study. Based on the type of lung transplantation, cases were categorized as either single lung transplantation or bilateral lung transplantation. All of the patients were operated using a transverse incision called a clamshell incision which allows greater access than the traditional sternotomy or thoracotomy. Intraoperative data, including the duration of the transplant procedure, the quantity of blood products transfused, and the utilization of intraoperative extracorporeal membrane oxygenation (ECMO), were documented. The duration of postoperative intensive care unit (ICU) and hospital stays for each recipient were calculated. After the operation, the history of ECMO use, hemodialysis requirement, and occurrence of primary graft dysfunction (PGD) was documented for each case. The PGD status was additionally confirmed through radiographs and blood gas results found in the recipients’ files. An intubation duration exceeding >5 days, the necessity for re-intubation following extubation, or the requirement for tracheostomy was categorized as prolonged extubation and documented for each patient. Recipients underwent pulmonary function tests within a minimum of two months after the operation to monitor graft function. The average of the two highest consecutive post-transplant FEV_1_ values, taken at intervals of at least three weeks, was considered the maximum FEV_1_ level (best FEV_1_). This value, along with the corresponding time in months, was determined as the point of best FEV_1_ measurement. A reduction of ≥20% in FEV_1_ after achieving the best FEV_1_ value was classified as chronic rejection, specifically chronic lung allograft dysfunction (CLAD), after excluding any other potential causes.

Demographic information of COPD recipients, as well as operative and post-operative clinical data, have been compiled and presented by gathering all relevant data. To investigate the effect of the BODE score on transplant results, a cut-off value was determined for each BODE component that could indicate the severity of the disease. The impact of the designated cutoff values for each component on intraoperative and postoperative clinical outcomes was examined and subsequently shared. The cut-off values determined for each BODE component are as follows: 20% for FEV_1_, 18 kg/m^2^ for BMI, 250 m for 6MWT.

### Statistical Methods

Data were encoded and recorded using IBM SPSS for Windows version 22.0 software (SPSS Inc., Chicago, IL, USA). Descriptive results are presented as frequencies and percentages for categorical variables. The variables were assessed visually (using probability plots and histograms), and the Shapiro-Wilk test showed that the distribution was non-parametric. Numerical variables are expressed as medians and minimum-maximum ranges. The Mann-Whitney-U test was carried out to compare the quantitative values of two independent groups due to the non-parametric distribution. The Chi-square test was used in group comparisons of nominal variables. All analyses were performed as two-sided hypotheses with a 5% significance level and a 95% confidence interval. A 2-tail p< 0.05 was considered statistically significant.

## RESULTS

We conducted an analysis of the clinical data of lung transplant recipients with COPD. This analysis encompassed

1) Demographic information,

2) Intraoperative data, and

3) Short- and long-term clinical outcomes, with a specific focus on the comparison between single and double transplants. Finally, we investigated the effect of BODE index components, on each set of posttransplant clinical data.

During the study period, 90 lung transplants were performed of which 34 (37.8%) were for COPD. Of the COPD patients, 2 (5.8%) were female and 3 (8.8%) had single transplants. The median age of transplant recipients with COPD was 57 (34-69) years. The median LAS before transplantation was 34.35 (31.90-45.44) and the median BMI was 21.3 (15.5-29.0) kg/m^2^. The data for COPD patients are summarized in Table 2 .

As one patient passed away during the operation, data other than demographic characteristics were analyzed for 33 patients. A median of two units (range= 0-10) of erythrocyte suspension (ES) and 10 units of fresh frozen plasma transfusion were required for each procedure. Intra-op and post-op ECMO were needed for 11 and four patients, respectively. The median duration of mechanical ventilation after the surgery was 24 hours and 6 (18.1%) patients needed prolonged extubation. The median duration of ICU and hospital stay was 12 and 29 days, respectively. Table 3 shows the distribution of post-op data in single and double lung transplants.

Out of the total recipients, 12 (36.3%) developed primary graft dysfunction (PGD), suggesting a predisposition for PGD in the context of single lung transplants. Post-op median best FEV_1_ ratio was higher in double transplants. All 5 CLAD events occurred in double transplants. Table 4 represents the clinical outcomes of single and double transplants.

Since March 2013, over a median follow-up period of 43 months (ranging from 1 to 117 months), hypertension, diabetes mellitus, chronic renal failure (without hemodialysis), CLAD, and malignancy have developed in 24, 12, 13, 5, and 2 recipients, respectively.

We also investigated the influence of specific components of the BODE index on clinical outcomes following transplantation, including intra-op ECMO use, post-op ECMO use, prolonged extubation, PGD, days on mechanical ventilation, ICU stay, hospital stay, and CLAD (at any stage). In this study, each BODE component (FEV_1_ with a cut-off of 20% predicted, 6MWT with a cutoff of 250 m, BMI with a cut-off of 18 kg/m²) had no significant impact on postoperative clinical outcomes. However, unlike the BODE components, when analyzed for age, intraoperative ECMO use (p= 0.030) prolonged extubation (p= 0.042), PGD (0.012) and CLAD cases (p= 0.078) were interestingly higher in recipients who were ≤60 years of age than ˃60.

**Table 2:** Demographics of COPD recipients

Parameters	Value
Recipients (n)	34
SLuTx/DLuTx (n)	3/31
Follow-up time (months; median, min-max)	43 (1-117)
Male/Female (n)	32/2
Age (years; median, min-max)	57 (34-69)
Height (cm; median, min-max)	167 (154-186)
Weight (kg; median, min-max)	63 (42-85)
BMI (kg/m^2^; median, min-max)	21.3 (15.5-29.0)
6-MWT (meters; median, min-max)	180 (20-450)
BODE score (number; median, min-max)	8 (5-10)
PAP (mmHg; median, min-max)	25 (17-60)
LAS (number; median, min-max)	34.35 (31.90-45.44)

BMI: Body-mass index, DLuTx: Double lung transplantation, LAS: Lung allocation score PAP: Pulmonary arterial pressure, SLuTx: Single lung transplantation, 6MWT: Six-minute walking test.

**Table 3:** Demographics of COPD recipients

Parameters	SLuTx recipients (n= 3)	DLuTx recipients (n= 30)	All recipients (n= 33)
Age (years; median, min-max)	56 (55-69)	61 (34-64)	57 (34-69)
Intra-op erythrocyte suspension transfusion (unit; median, min-max)	2 (1-4)	2 (0-10)	2 (0-10)
Intra-op frozen plasma transfusion (unit; median, min-max)	4 (4-6)	10 (5-18)	10 (4-18)
Intra-op ECMO (patient)	1	10	11
Post-op ECMO (patient)	-	4	4
ICU time (days; median, min-max)	9 (6-13)	12 (3-80)	12 (3-80)
Hospitalization time (days; median, min-max)	24 (22-32)	30 (3-92)	29 (3-92)
Mechanical ventilation time (hours; median, min-max)	23 (18-96)	24 (10-1680)	24 (10-1680)
Prolonged extubation (patient)	0	6	6

DLuTx: Double lung transplantation, ECMO: Extracorporeal membrane oxygenation, ICU: Intensive care unit, SLuTx: Single lung transplantation.

**Table 4:** Post-transplant clinical results

	SLuTx recipients (n= 3)	DLuTx recipients (n= 30)	All recipients (n= 33)
Follow-up time (months; median, min-max)	57 (4-61)	43 (1-117)	43 (1-117)
PGD (number of patients)	3	9	12
Pre-op FEV_1_ (%; median, min-max)	19 (9-20)	20 (11-35)	20 (9-35)
Best FEV_1_ (%; median, min-max)	60 (58-82)	94 (65-126)	82 (58-126)
Best FEV_1_ time (month; median, min-max)	11 (10-12)	12 (5-18)	12 (5-18)
CLAD (any stage, patient)	-	5	5
CLAD time (months; median, min-max)	-	30 (10-58)	30 (10-58)

CLAD: Chronic lung allograft dysfunction, DLuTx: Double lung transplantation, FEV_1_: Forced expiratory volume in first second, PGD: Primary graft dysfunction, SLuTx: Single lung transplantation.

## DISCUSSION

In our study, which investigated the outcomes of lung transplantation in COPD patients, over 90% of COPD recipients underwent double transplants. The post-transplant clinical outcomes of both single and double transplants demonstrated comparable data. The impact of pre-transplant BODE components on various post-transplant clinical outcomes was also examined; however, no statistically significant associations were identified. One of the interesting findings from our study is that recipients under the age of 60 experience higher post-transplant morbidity compared to those who are 60 years or older.

In the early 1980s, when lung transplantation was first carried out successfully, single lung transplantation was favored due to its lower surgical morbidity. However, today, double lung transplantation has gained preference owing to its superior long-term outcomes ( 12 , 13 ). Nevertheless, single lung transplantation remains a reasonable option in certain cases. It has been noted that for COPD patients over 60 years of age, single lung transplantation is recommended to mitigate additional surgical morbidity ( 14 , 15 , 16 ). In our study, our goal was to conduct double lung transplantation whenever the patients’ clinical conditions permitted. We opted for a single lung transplantation in one case, to lower surgical morbidity in a 69-year-old patient. In the two remaining cases, the decision to perform single lung transplantation was made for patients under 60 years of age due to unforeseen technical challenges that necessitated the surgical team to choose single transplants ( 17 ).

In this study, we investigated the impact of pretransplant demographic and clinical variables of recipients on both intraoperative and postoperative outcomes among COPD recipients. It was observed that there were comparable operation times, durations of mechanical ventilation, periods of intensive care stay, and overall hospital stays when comparing single and double lung transplants. As a result, our findings regarding surgical morbidity did not identify a significant clinical distinction between single and double transplants.

The incidence of PGD, which is one of the early complications of lung transplantation, was significantly higher in single lung recipients compared to double lung transplants. The elevated occurrence of PGD in single lung transplants could be hypothesized as a physiological consequence of native lung hyperinflation (NLH) impacting the allograft ( 16 ). Moy et al. and Weill et al. reported that NHL occurs in up to 20% of single transplants ( 18 , 19 ). The causative relationship between NLH and PGD remains a subject of debate: whether NLH leads to clinical PGD by altering the position and compression of the allograft, or if PGD occurring in the allograft diminishes its inflation, resulting in NLH within the native lung.

As anticipated, double lung transplants demonstrated higher FEV_1_ values in the long term ( 20 , 21 ). However, this outcome did not attain statistical significance, potentially attributed to the limited number of cases. Latos et al. reported that after one year, FEV_1_ increased to 80% of expected in DLuTx recipients and to 55% in SLuTx recipients. In our study, recipients achieved a maximum FEV_1_ value of 94% of the expected for double lung transplantation (DLuTx) and 60% of the expected for single lung transplantation (SLuTx), without any temporal restrictions ( 22 ).

Moreover, earlier research has pointed out that single lung transplants have drawbacks concerning CLAD, a key reason for graft failure in long-term lung transplant recipients ( 23 , 24 ). Pochettino et al. noted that there is no difference between single lung transplants (SLuTx) and double lung transplants (DLuTx) in terms of CLAD ( 25 25 ). Similarly, a study by Meyer et al. revealed no disparity between single and double transplants regarding CLAD development after a three-year follow-up ( 14 ). However, Hadjiliadis et al. reported that DLuTx offers freedom from CLAD at three and five years post-transplant ( 23 , 24 ). A noteworthy outcome of our study was the detection of all 5 CLAD cases among DLuTx recipients, even though DLuTx cases had a shorter median follow-up time compared to SLuTx recipients (57 months for SLuTx, 43 months for DLuTx).

The BODE score is recommended by ISHLT as a predictor for COPD recipient candidate selection ( 11 ). Numerous studies have examined the impact of the BODE score on post-transplant survival ( 26 , 27 , 28 ). Our study, however, aimed to establish specific cutoff values for each BODE index component and assess the influence of these components on distinct post-transplant clinical outcomes, to identify a marker that could guide clinicians during the posttransplant follow-up phase. In terms of these cut-off values, the BODE components did not have a significant clinical effect. Recipients with better 6MWT results at the time of listing had shorter hospital stays, in line with previous studies, but it was not statistically significant ( 26 ). FEV_1_ value and BMI at the time of listing did not have a significant impact on post-op outcomes. An intriguing discovery was that recipients with a BMI below 18 required more intra-operative ECMO yet experienced a shorter duration of mechanical ventilation. However, neither of these findings reached statistical significance.

The impact of recipient age on postoperative outcomes was also investigated. Interestingly, recipients aged 60 years or younger exhibited a higher occurrence of intraoperative ECMO utilization, prolonged extubation, and PGD, and these differences were statistically significant (p< 0.05). Additionally, as highlighted in a previous report, CLAD was more prevalent in recipients aged 60 years or younger, although this did not reach statistical significance (p= 0.078) ( 29 ). However, in the ISHLT document, a recipient age over 60 years is identified as a risk factor for CLAD ( 30 , 31 ). Notably, in our two patients aged 60 years or younger, CLAD was induced by lung injury resulting from severe coronavirus disease-19 ( 32 , 33 ). The limited number of cases may explain the observed frequency of CLAD in recipients under 60 years of age. It is important to note that these findings should be validated with a larger cohort of cases.

Our study has limitations due to its retrospective design and a small number of patients. Notably, our study is the first in our country to report outcomes for COPD patients following lung transplantation. Furthermore, our findings challenge some established ideas in the literature regarding the relationship between age and lung transplantation outcomes.

In summary, our study aimed to explore how prelisting patient data influences post-transplant outcomes for individuals with COPD undergoing lung transplantation. The study revealed no disparities in surgical complications between single and double transplants, and no substantial impacts on short- and long-term outcomes were observed when assessing individual BODE components. However, an interesting divergence from the literature emerged when comparing COPD recipients aged 60 years or younger with those over 60, revealing unexpected insights. We believe that it is crucial to assess and confirm these findings in a larger patient cohort.

## Ethical Committee Approval

This study was approved by Ankara City Hospital Clinical Researches No 1 Ethics Committee (Decision no: E1-22, 3134, Date: 28/12/2022).

## CONFLICT of INTEREST

The authors declare that they have no conflict of interest.

## AUTHORSHIP CONTRIBUTIONS

Concept/Design: ST, EY

Analysis/Interpretation: All of authors

Data acqusition: ST, MB, FŞ, MAB, EY

Writing: ST, EY

Clinical Revision: ST, FÇB, EY

Final Approval: ST, EY
